# Post-Traumatic Stress and School Adaptation in Adolescent Survivors Five Years after the 2010 Yushu Earthquake in China

**DOI:** 10.3390/ijerph16214167

**Published:** 2019-10-29

**Authors:** Shou Liu, Li Lu, Zheng-Zhong Bai, Min Su, Zheng-Qing Qi, Shi-Yu Zhang, Yuan Chen, Bing-Yu Ao, Feng-Zhen Cui, Emmanuel Lagarde, Kehshin Lii

**Affiliations:** 1 Department of Public Health, Medical College, Qinghai University, Qinghai 810001, China; 2 Department of Statistics, University of California, Riverside, 337 Olmsted Hall, UCR, Riverside, CA 92521, USA; 3 Team IETO, Bordeaux Population Health Research Center, UMR U1219, INSERM, Université de Bordeaux, 33076 Bordeaux, France; 4 School of Public Administration, Inner Mongolia University, Inner Mongolia 010021, China

**Keywords:** adolescent, post-traumatic stress disorder (PTSD), school adaptation, Tibetan, Yushu earthquake

## Abstract

(1) Background: The devastating Ms 7.1 earthquake struck Yushu city, China, in 2010, leading to serious consequences and damage in the central Tibetan Plateau. This study aimed to assess school adaptation and post-traumatic stress disorder (PTSD) symptoms of adolescent survivors five years after the Yushu earthquake. (2) Methods: A large-scale, school-based mental health survey was conducted 5 years after the earthquake among Tibetan students in the city of Yushu using the Adolescent’s School Adaptation Scale (ASAS) and the PTSD Checklist. (3) Results: A total of 1976 questionnaires were collected. A total of 30.7% of Tibetan adolescents had poor school adaptation and 19.5% were estimated as having probable PTSD. Logistic regression showed that females (OR = 0.73, 95% CI: 0.60–0.89), senior students (OR = 0.48, 95% CI: 0.39–0.59), and those who participated in post-disaster reconstruction (OR = 0.68, 95% CI: 0.54–0.85) were less likely to have poor school adaptation, while a positive association was observed among those buried under a collapsed building (OR = 1.47, 95% CI: 1.04–2.09) and those who experienced bereavement (OR = 1.77, 95% CI: 1.27–2.45). Students who had experienced bereavement were also more likely to have PTSD (OR = 1.60, 95% CI: 1.12–2.28). (4) Conclusions: The post-traumatic effects of the Yushu earthquake on Tibetan adolescents were severe and long-lasting. Sustainable long-term mental health services to help adolescents to restructure their mental health are necessary.

## 1. Introduction

The city of Yushu is the capital the Tibetan Autonomous Prefecture of Yu Shu and located in the southwest Qinghai province of China at an average altitude of 4493.4 meters with 100,000 inhabitants. The city was hit by an earthquake (Ms 7.1) in 2010, which caused the death of 2698 people and injured 12,135 [1,2]. It has been estimated that a minimum of 85% of houses were destroyed in Yushu county, almost all (99%) houses collapsed in the most badly affected village, and 70% of school buildings collapsed [1]. 

The earthquake broke down basic social infrastructures and had a profound impact on various aspects of the quality of life of local residents [3,4]. In the early post-disaster reconstruction period, local government and research institutions focused primarily on providing emergency medical services and coping with stress disorders for earthquake survivors [5,6]. In addition, local healthcare services established outreach mental health services in collaboration with social workers in schools and medical institutions.

Several studies have assessed individuals’ mental health and traumatic symptoms following the earthquakes [7,8,9], where the detrimental psychological effects of earthquakes often persist for a decade or longer [10]. Previous studies have identified the associations between post-traumatic stress disorder (PTSD) symptoms, anxiety, and depression and the experience of the earthquake [1,8,11,12]. Sleep disorders have also been reported by earthquake survivors [13,14]. A study conducted among 2080 survivors in Wenchuan found that although the prevalence of PTSD, anxiety, and depression disorders decreased over time after the earthquake, its effect on the mental health remained severe one year later, with a 40.1% prevalence of PTSD [15]. Another study showed that the prevalence of PTSD was 44.5% among 278 Lushan survivors 13 to 16 months after the earthquake [16]. 

Large-scale screening programs to assess the extent of post-disaster psychiatric disorders among adolescents have been continuously conducted [2,3,17]. The family environment was reportedly associated with adolescents’ PTSD [18]. Studies conducted on adolescents after the Yushu earthquake were all related to PTSD [19,20]; no studies have been so far conducted on the adolescents’ school adaptation. In literature, school adaptation difficulties were described among sexually abused girls [21], and longitudinal studies showed that stress significantly decreases subsequent school performance and completion [22,23]. The issue of adolescents’ school adaptation and adjustment in the context of a strong earthquake therefore needs to be investigated. Due to the population composition of the Tibetan Autonomous Prefecture of Yu Shu, Tibetans account for 98% of the total population [24]. Survivors in Yushu earthquake are known to be Tibetan and have a belief in Tibetan Buddhism, where the belief could affect various aspects of adolescent survivors’ social and daily life [2], such as the school adaptation and perspectives on the loss of family members, etc.

The present study aimed to assess the 5-year consequences and associated factors of the 2010 Yushu earthquake on school adaptation and PTSD symptoms of Tibetan adolescents. Based on findings from previous post-earthquake studies, we made the hypothesis that the school maladaptation and PTSD symptoms among student survivors 5 years after the Yushu earthquake would still be common.

## 2. Materials and Methods

### 2.1. Study Design, Participants, and Data Collection

A large-scale, school-based mental health survey was conducted on Tibetan students/adolescents attending the Tibetan boarding school (The Second Middle School of Yushu) in Yushu city between June and September 2015. There are two boarding middle schools in Yushu city, where students coming from different areas of the city including the remote areas generates a diversified group of students in this school, which guarantees the representativeness of the sample. Tibetan middle school students who survived the Yushu earthquake were included using a cluster-sampling method. Due to the objective stating that we only focus on the symptoms of minority students, Han ethnic students were excluded from this study. Twenty students were randomly selected for a pilot survey and standardized answers were generated. School teachers were trained to introduce the study and administer the questionnaires. 

Teachers introduced the objectives and procedure of the survey to all students before distributing questionnaires in each class and all students were informed of the principle of anonymity and voluntary participation regarding the survey. The teacher distributed the questionnaires to the entire class and the students were asked to return their questionnaires after completing it within approximately 30 min. All students involved verbally agreed to participate in the survey and written informed consent was provided by their guardians in accordance with local ethical regulations for epidemiological surveys. 

### 2.2. Assessment Tools

The questionnaire was divided into three parts: (1) socio-demographic characteristics (gender, grade, and adolescents’ different earthquake experiences, e.g., injury, injury of a family member, buried under debris, bereavement, property damage, living in the hardest-hit area, and involved in post-disaster reconstruction; (2) Adolescent’s School Adaptation Scale (ASAS); and (3) PTSD symptoms. For consistency, the original Chinese questionnaires were first translated into the Tibetan language and then translated back into Chinese by different Tibetan experts to compare the differences between the translations and modify the Tibetan translation accordingly.

The Adolescent’s School Adaptation Scale (ASAS) was used to assess the participants’ school adaptability five years after the earthquake, where the Cronbach alpha of the scale was 0.78 [25]. Prior to the formal investigation, a preliminary survey was conducted in Tibetan adolescents to evaluate the measuring quality of this scale. The test–retest reliability and content validity of the scale among the samples were 0.82 and 0.89, respectively. Test–retest reliability is the correlation coefficient between the results of a repeat measurement on the same participants (0.82) and the content validity is the consistency between participants’ understanding of, and answer to, the question and what the item designer wanted to ask was 0.89 (content validity ratio). The ASAS assessed eight subtypes of adaptability by using 54 items: (a) stress from the teacher (7 items), (b) principle breach (10 items), (c) academic anxiety (11 items), (d) sense of justice (7 items), (e) peer relationships (6 items), (f) school attractiveness (4 items), (i) competition awareness (5 items), and (j) teacher–student relationship (4 items). There were five possible responses to each item from 1 (“completely agree”) to 5 (“completely disagree”). Poor adaptability for a given subtype was defined as the average score of the corresponding items being greater than or equal to 3. Overall poor school adaptability was defined as the average score of the all items being greater or equal to 3 [25]. 

The Post-Traumatic Stress Disorder (PTSD) Checklist was used to assess the PTSD [26]. It is a widely used self-reported scale [2,27], and the Chinese version has been validated with good psychometric properties (Cronbach’s alpha above 0.77) [28]. It consists of 17 items and represents the three following dimensions: (a) re-experiencing symptoms (5 items), (b) avoidance (7 items), and (c) hyper-arousal (5 items). Each symptom was self-assessed on a five-point Likert scale ranging from 1 ("not at all”) to 5 ("extremely”). Adolescents with a score ≥44 were considered to have PTSD and a higher score represented severe symptoms [2,28]. 

### 2.3. Data Analysis

Independent-sample *t*-tests and chi-square (χ^2^) tests were used for the univariate analyses. Logistic regression analyses were conducted to explore the associations by setting the ASAS and PTSD as dependent variables separately. All predictors in preliminary analyses were considered as independent variables. A *p*-value less than 0.05 was considered statistically significant. All statistical procedures were completed using STATA version 12.0 (Stata Corporation, College Station, TX, USA).

### 2.4. Ethics Approval

This study was approved by the the principals of Yushu Second Prefecture Middle School and The Ethnic Committee of the Medical College of Qinghai University, authorization number: QHMC-2015-00001-PH.

## 3. Results

### 3.1. Basic Characteristics of the Participants

A total of 2000 questionnaires were distributed and collected with a response rate of 100%. Questionnaires from six students from the Han ethnic group and 18 from students who did not experience the earthquake were excluded. Therefore, 1976 (98.8%) collected questionnaires were used in this study with 1146 (58.0%) female and 634 (32.1%) junior students. The basic demographic characteristics and traumatic experiences of the students are presented in Table 1. The proportion of participants who reported personal injury, injury of a family member, burial under debris, bereavement, and property damage were 15.5%, 17.8%, 9.0%, 11.5%, and 31.3%, respectively. The participants who were living in the hardest-hit area were 32.5%, and 35.8% were involved in post-disaster reconstruction.

### 3.2. Predictors of School Maladaptation

The average ASAS score was 21.58 (SD = 2.74) among the 1976 participants, ASAS subtypes with the highest average scores were stress from the teacher (3.27; SD = 0.60), academic anxiety (3.25; SD = 0.45), and competition awareness (2.71; SD = 0.58). The prevalence of poor school adaptability was 30.7% (*n* = 606, 95% CI: 28.6%–32.8%). Social demographic characteristics of school adaptation and its subtypes are shown in Table 2 and Table S1, respectively. 

Logistic multivariate regression showed that female (OR = 0.73, 95% CI: 0.60–0.89, *p* = 0.002) and senior (OR = 0.48, 95% CI: 0.39–0.59, *p* < 0.001) students, and those who where involved in post-disaster reconstruction (OR = 0.68, 95% CI: 0.54–0.85, *p* = 0.001), were less likely to have school maladaptation, while those who were buried under a collapsed building (OR = 1.47, 95% CI: 1.04–2.09, *p* = 0.03) and suffered bereavement (OR = 1.77, 95% CI: 1.27–2.45, *p* = 0.001) had a higher risk of poor school adaptation. PTSD symptoms showed weak evidence for a positive relationship with overall school maladaptation (OR = 0.78, 95% CI: 0.60–1.003, *p* = 0.053). The results of the logistic regressions on the associations between independent variables and the prevalence of poor school adaptation are shown in Table 3. 

### 3.3. Predictors of PTSD

The overall average PTSD score was 41.20 (SD = 10.98), and the average scores for re-experiencing symptoms, persistent avoidance symptoms, and hyper-arousal symptoms were 12.38 (SD = 3.87), 16.67 (SD = 4.94), and 12.15 (SD = 4.16), respectively. There were 19.5% (*n* = 386, 95% CI: 17.8%–21.4%) of adolescents who were positive for PTSD. The univariate analyses of PTSD predictors are shown in Table 4. 

Multivariate logistic regression suggested that participants who experienced bereavement (OR = 1.60, 95% CI: 1.12–2.28 *p* = 0.009) were more likely to report PTSD (Table 5).

## 4. Discussion

Exposure to a range of traumatic events can cause detrimental consequences for survivors’ mental health and wellbeing [29,30]. This study identifies that the prevalence of poor school adaptation among Tibetan adolescents 5 years after the 2010 Yushu earthquake was 30.7%, and 19.5% of adolescents met the criteria on the PTSD checklist. Of note, the interventions for post-traumatic stress must be carried out in children and adolescents 2–6 months after the traumatic events due to the deterioration of the condition and decreased effectiveness of the therapy interventions [31]. However, it is also important to evaluate the long-term influence of the traumatic event from an epidemiological viewpoint.

Poor school adaptation was common among the participants in our study. Although a wide range of psychiatric disorders have been described previously among adolescents who experienced a disaster [17], the prevalence of poor school adaptation after an earthquake has been rarely explored so far. One clinic-based study identified that 54% of sexually abused girls presented disabilities in school adaptation, which was higher than our figure [21]. Contrary to our study, which examined eight subscales, the authors used some of the variables from the Child Behavior Checklist [32] to assess three domains of school adaptation, i.e., academic performance, social functioning, and externalized behaviors [21]. Another previous study identified that school non-completion were more frequent among those students who had more life-changing events and higher level of stress [23]. Further works are clearly needed to describe the vulnerability to school adaptation and dropping out induced by stressful life events. 

Males, junior students, and those who did not participate in post-disaster reconstruction were more likely to experience poor school adaptation after the earthquake over the long term. Females were found more prone to report psychological effects after an earthquake in other studies [15,18], although school adaptation was rarely studied. One cohort study found that younger children were more likely to report PTSD symptoms following the 1999 Athens earthquake, probably because younger children may be less able to deal with traumatic experiences than those who are older, both emotionally and cognitively [9]. Participating in post-disaster reconstruction mainly referred to the activities within the adolescents’ power, such as participating in the cleanup of the ruins, rearranging the home, etc., which provided adolescents with the chance to integrate into group activities and benefit from more social support, which in turn could help them to develop positive attitudes toward life and offset the negative effects of traumatic events on adaptation [22]. Those who have been buried under a collapsed building and who experienced bereavement were more likely to report poor school adaptation. With respect to PTSD, other studies unsurprisingly evidenced that people who experienced severe traumatic events are more likely to suffer from PTSD [2]. There was weak evidence on the inverse association between PTSD and school maladaptation. One possible explanation is that those with PTSD sought or accepted more social support in the hope of alleviating their suffering [33]. However, the content of our study did not provide information on social support and the hypothesis cannot be tested.

Published studies on PSTD after earthquakes reported a wide range of PCL scores and prevalence. The scores of our participants were higher than another study that also focused on Tibetan adolescents 4 years after the earthquake (36.8, SD = 12.1), while the prevalence between their study and ours was similar (19.3%) [2]. Much higher prevalences have been reported elsewhere: 37.5% among high school students 10 months after an earthquake in L’Aquila, Italy [7]; and 20.9% among women adults (who were pregnant during or after the earthquake) 4 years after the Wenchuan earthquake in China [34]; and 33.7% among the survivors who were in the hard-hit areas 3–4 months after the Yushu earthquake [1]. Almost 65% of Pakistani earthquake survivors met the criteria for probable PTSD 3 years after the initial shock [35]. The figure in our study was over twice as high as those reported among Taiwanese survivors 6 months after the Chi-Chi earthquake in 1999 (7.9%) [36]. The difference in published studies may be due to contextual factors, such as regional socio-economical differences, assessment and diagnostic methods, or assessment tools with differences in terms of cut-off values, time points, the degree of traumatic exposure, destructive extent of the events, etc. [1,29,35].

Adolescents who had experienced bereavement due to the earthquake were more likely to be positive for PTSD, those suffered an injury also showed weak evidence of a positive association. Even though the prevalence of PTSD varies widely among studies, personal injury and a sudden and/or violent loss of a loved or closed person during a natural disaster have been repeatedly reported to have an adverse influence on individuals’ mental health, such as resulting in PTSD [37,38,39]. For example, survivors who were injured during the 2004 tsunami in Tamil Nadu, India, were approximately 3 times as likely to report PTSD [40]. Bereavement experience was also reported to be positively correlated with PTSD among high school girls who survived the tsunami [41]. Children who had lost family members after a 7.4-magnitude earthquake in Turkey were more likely to have PTSD with comorbid depression [11]. Previous studies reported that among the earthquake survivors, PTSD was more common among women, those with less social support, and those with low income [1,7,42], which was not confirmed in our study. 

Treatment and intervention programs of traumatized adolescents should be launched in view of the long-lasting effects of the earthquake. Integrated efforts from the family, school, community, and society may serve to provide a collaborative trauma systems therapy (TST) approach for adolescents [43,44]. The direct care staff members in our study are the teachers that adults spend the most time with [44]; therefore, training regarding the ability to recognize adolescents’ emotional state and relevant coping methods could be performed among teachers. In addition, arts-based mindfulness group activities that can enhance the sense of collective cohesion and teamwork [45] and a rotating appointment of class cadre roles that could reinforce and expand a person’s conception of responsibility [46] are supposed to be effective and could help to restructure adolescents’ mental health. 

This study explored factors associated with poor school adaptation and PTSD among adolescents 5 years after the earthquake, which could provide government and related institutions with the evidence to implement long-term intervention programs for school-aged populations who have experienced traumatic events in China. However, there are some limitations in this study. First, the participants were recruited from a single school rather than from several randomly selected schools, which may limit the generalizability of the results. Second, some potentially relevant information, such as social support, economic level, etc., was not collected in our study. In addition, the scales used in this study were not validated in the Tibetan language, and the ASAS scale was not nationally validated and commonly used, which made it impossible to make comparisons between our results and those from other studies. Finally, due to the exploratory nature of the present study, the causality between possible factors and poor school adaptation and PTSD cannot be established.

## 5. Conclusions

In conclusion, the psychological and academic consequences of the Yushu earthquake on Tibetan adolescents were severe and long-lasting, underscoring the need to provide sustainable mental health services and intervention programs to help adolescents restructure their mental health after the disaster. Further studies and therapy interventions should be performed after the traumatic events to avoid the conversion of related disadvantages from acute to chronic status. 

## Figures and Tables

**Table 1:** Demographic characteristics and traumatic experience among adolescent survivors of the Yushu earthquake, China (*N* = 1976).

Title	Total (%)	Grade		*χ*^2^ (*p*)	Gender		*χ^2^ (p)*
		Junior (*n* = 634)	Senior (*n* = 1342)		Male (*n* = 830)	Female (*n* = 1146)	
Injury (Yes)	307 (15.5)	106 (16.7)	201 (15.0)	0.99 (0.32)	152 (18.3)	155 (13.5)	8.41 (**0.004**)
Injury of family member (Yes)	353 (17.8)	112 (17.7)	240 (17.9)	0.01 (0.91)	146 (17.6)	206 (18.0)	0.05 (0.83)
Buried under debris (Yes)	178 (9.0)	57 (9.0)	121 (9.0)	0.0004 (0.99)	87 (10.5)	91 (7.9)	3.79 (0.051)
Bereavement (Yes)	228 (11.5)	57 (9.0)	171 (12.7)	5.94 (**0.015**)	87 (10.5)	146 (12.7)	3.86 (**0.049**)
Property damage (Yes)	619 (31.3)	199 (31.4)	420 (31.3)	0.002 (0.97)	260 (31.3)	359 (31.3)	<0.001 (1.00)
Living in hardest-hit area * (Yes)	643 (32.5)	211 (33.3)	432 (32.2)	0.23 (0.63)	264 (31.8)	379 (33.1)	0.35 (0.55)
Involved in post-disaster reconstruction (Yes)	708 (35.8)	205 (32.3)	503 (37.5)	4.96 (**0.03**)	297 (35.8)	411 (35.9)	0.001 (0.97)
Total (%)	1976	634 (32.1)	1342 (67.9)	-	830 (42.0)	1146 (58.0)	-

* Adolescents living within 60 km of the epicenter. The differences in the proportion of the traumatic experiences were analyzed according to different gender and grade groups. Bold values: *p* < 0.05.

**Table 2:** Univariate analyses of ASAS among adolescent survivors.

Variables	Good School Adaptation (*n* = 1370)		Poor School Adaptation (*n* = 606)		Statistics	
	*n*	%	*n*	%	*χ* ^2^	*p*
Gender (Female)	828	60.4	318	52.5	10.94	**0.001**
Grade (Senior)	999	72.9	343	56.6	51.35	**<0.001**
Injury	193	14.1	114	18.8	7.15	**0.008**
Injury of a family member (Yes)	222	16.2	130	21.5	7.90	**0.005**
Buried under debris (Yes)	106	7.7	72	11.9	8.80	**0.003**
Bereavement (Yes)	135	9.9	93	15.3	12.42	**<0.001**
Property damage (Yes)	435	31.8	184	30.4	0.38	0.54
Living in hardest-hit area (Yes)	451	32.9	192	31.7	0.29	0.59
Involved in post-disaster reconstruction (Yes)	524	38.2	184	30.4	11.36	**0.001**
PTSD (Yes)	278	20.3	108	17.8	1.63	0.20

Note: ASAS: Adolescent’s School Adaptation Scale; PTSD: Post-Traumatic Stress Disorder. Bold values: *p* < 0.05.

**Table 3:** Logistic regression analyses on the associations between independent variables and school maladaptation (*N* = 1976).

Variables	OR	*p*	95% CI	
			Lower	Upper
Gender (Female)	0.73	**0.002**	0.60	0.89
Grade (Senior)	0.48	**<0.001**	0.39	0.59
Injury (Yes)	1.28	0.093	0.96	1.72
Injury of a family member (Yes)	1.29	0.088	0.96	1.73
Buried under debris (Yes)	1.47	** 0.030**	1.04	2.09
Bereavement (Yes)	1.77	**0.001**	1.27	2.45
Property damage (Yes)	0.85	0.204	0.67	1.09
Living in hardest-hit area (Yes)	0.84	0.171	0.66	1.08
Involved in post-disaster reconstruction (Yes)	0.68	**0.001**	0.54	0.85
PTSD (Yes)	0.78	0.053	0.60	1.003

Adjusted R^2^ = 0.044. ASAS: Adolescent’s School Adaptation Scale; PTSD: Post-Traumatic Stress Disorder. Bold values: *p* < 0.05.

**Table 4:** Univariate analyses of PTSD among adolescent survivors.

Variables	Without PTSD (*N* = 1590)		With PTSD (*N* = 386)		Statistics	
	***n***	**%**	***n***	**%**	***χ*^2^**	***p***
Gender (Female)	926	58.2	220	57.0	0.20	0.66
Grade (Senior)	1080	67.9	262	67.9	0.0003	0.99
Injury (Yes)	231	14.5	76	19.7	6.30	**0.01**
Injury of a family member (Yes)	269	16.9	83	21.5	4.46	**0.04**
Buried under debris (Yes)	132	8.3	46	11.9	4.95	**0.03**
Bereavement (Yes)	164	10.3	64	16.6	11.95	**<0.001**
Property damage (Yes)	496	31.2	123	31.9	0.06	0.80
Living in hardest-hit area (Yes)	511	32.1	132	34.2	0.60	0.44
Involved in post-disaster reconstruction (Yes)	570	35.8	138	35.8	0.001	0.97

PTSD: Post-Traumatic Stress Disorder. Bold values: *p* < 0.05.

**Table 5:** Logistic regression analyses on the associations between independent variables and PTSD (*N* = 1976).

Variables	OR	*p*	95% CI	
			Lower	Upper
Gender (Female)	0.96	0.697	0.76	1.20
Grade (Senior)	0.99	0.918	0.78	1.26
Injury (Yes)	1.29	0.120	0.94	1.78
Injury of a family member (Yes)	1.10	0.583	0.79	1.52
Buried under debris (Yes)	1.28	0.205	0.87	1.88
Bereavement (Yes)	1.60	**0.009**	1.12	2.28
Property damage (Yes)	0.87	0.340	0.66	1.15
Living in hardest hit area (Yes)	0.96	0.774	0.73	1.27
Involved in post-disaster reconstruction Yes)	0.94	0.636	0.73	1.21
PTSD (Yes)	0.96	0.697	0.76	1.20

Adjusted R^2^ = 0.09. ASAS: Adolescent’s School Adaptation Scale; PTSD: Post-Traumatic Stress Disorder. Bold values: *p* < 0.05.

## Supplementary Materials

The following are available online at https://www.mdpi.com/1660-4601/16/21/4167/s1, Table S1: Univariate analyses of ASAS and subscales among adolescent survivors (*N* = 1976).
