# Supplementary material for: Post-Traumatic Stress and School Adaptation in Adolescent Survivors Five Years after the 2010 Yushu Earthquake in China
Source: Int J Environ Res Public Health. 2019 Oct 29;16(21):4167. doi: 10.3390/ijerph16214167 (PMC6861989; doi:10.3390/ijerph16214167)
Supplement: Supplementary file 1 [file ijerph-16-04167-s001.pdf]

International Journal of
Environmental Research and Public Health

MDPI

Supplementary Material: Post-Traumatic Stress and School Adaptation in Adolescent Survivors Five Years after the 2010 Yushu Earthquake in China

Table S1. Univariate analyses of ASAS and subscales among adolescent survivors (N = 1976).

**Variables** *		**1** [a]	**2** [a]	**3** [a]	**4** [a]	**5** [a]	**6** [a]	**7** [a]	**8** [a]
		n **(%)**	***n*** **(%)**	***n*** **(%)**	***n*** **(%)**	***n*** **(%)**	***n*** **(%)**	***n*** **(%)**	***n*** **(%)**
**Gender**	Female (n = 1146)	796 (56.4)	353 (50.1)	839 (57.1)	209 (56.0)	265 (52.4)	230 (54.1)	405 (54.1)	214 (52.8)
	Male (n = 830)	616 (43.6)	351 (49.9)	630 (42.9)	164 (44.0)	241 (47.6)	195 (45.9)	343 (45.9)	191 (47.2)
**Grade**	Senior (n = 1342)	935 (66.2)	419 (49.5)	955 (65.0)	250 (67.0)	283 (55.9)	269 (63.3)	452 (60.4)	255 (63.0)
	Junior (n = 634)	477 (33.8)	285 (50.5)	514 (35.0)	123 (33.0)	223 (44.1)	156 (36.7)	296 (39.6)	150 (37.0)
**Injury**	No (n = 1673)	1188 (84.1)	569 (80.8)	1234 (84.0)	313 (83.9)	402 (79.4)	366 (86.1)	615 (82.2)	332 (82.0)
	Yes (n = 303)	224 (15.9)	135 (19.2)	235 (16.0)	60 (16.1)	104 (20.6)	59 (13.9)	133 (17.8)	73 (18.0)
**Injury of a family member**	No (n = 1633)	1159 (71.0)	562 (34.4)	1198 (73.4)	309 (18.9)	413 (25.3)	356 (21.8)	609 (37.3)	332 (20.3)
	Yes (n = 343)	253 (73.8)	142 (41.4)	271 (79.0)	64 (18.7)	93 (27.1)	69 (20.1)	139 (40.5)	73 (21.3)
**Buried under debris**	No (n = 1801)	1276 (90.4)	625 (88.8)	1330 (90.5)	335 (89.8)	447 (88.3)	393 (92.5)	674 (90.1)	368 (90.9)
	Yes (n = 175)	136 (9.6)	79 (11.2)	139 (9.5)	38 (10.2)	59 (11.7)	32 (7.5)	74 (9.9)	37 (9.1)
**Bereavement**	No (n = 1744)	1246 (88.2)	604 (85.8)	1292 (88.0)	325 (87.1)	445 (87.9)	373 (87.8)	647 (86.5)	351 (86.7)
	Yes (n = 232)	166 (11.8)	100 (14.2)	177 (12.0)	48 (12.9)	61 (12.1)	52 (12.2)	101 (13.5)	54 (13.3)
**Property damage**	No (n = 1360)	968 (68.6)	492 (70.0)	1000 (68.1)	287 (76.9)	352 (69.6)	314 (73.9)	511 (68.3)	275 (67.9)
	Yes (n = 616)	444 (31.4)	212 (30.0)	469 (31.9)	86 (23.1)	154 (30.4)	111 (26.1)	237 (31.7)	130 (32.1)
**Living in hardest-hit area**	No (n = 1333)	940 (66.6)	497 (70.6)	985 (67.1)	264 (70.8)	339 (67.0)	291 (68.5)	506 (67.6)	273 (67.4)
	Yes (n = 643)	472 (33.4)	217 (29.4)	484 (32.9)	109 (29.2)	167 (33.0)	134 (31.5)	242 (32.4)	132 (32.6)
**Involved in post-disaster reconstruction**	No (n = 1270)	913 (64.7)	468 (66.5)	939 (63.9)	262 (70.2)	344 (68.0)	303 (71.3)	502 (67.1)	286 (70.6)
	Yes (n = 706)	499 (35.3)	236 (33.5)	530 (36.1)	111 (29.8)	162 (32.0)	122 (28.7)	246 (32.9)	119 (29.4)
**PTSD**	No (n = 1590)	1187 (84.1)	584 (83.0)	1211 (82.4)	238 (63.8)	400 (79.1)	296 (69.6)	605 (80.9)	264 (65.2)
	Yes (n = 386)	225 (15.9)	120 (17.0)	258 (17.6)	135 (36.2)	106 (20.9)	129 (30.4)	143 (19.1)	141 (34.8)
**Total**		1412 (71.5)	704 (35.6)	1469 (74.3)	373 (18.9)	506 (25.6)	425 (21.5)	748 (37.9)	405 (20.5)

* Data of students using the ASAS are shown. a: 1—stress from teacher; 2—principle breach; 3—academic anxiety; 4—sense of justice; 5—peer relationship; 6—school attractiveness; 7—competition awareness; 8—teacher–student relationship. ASAS: Adolescent's School Adaptation Scale; PTSD: Post-Traumatic Stress Disorder.

© 2019 by the authors. Submitted for possible open access publication under the terms and conditions of the Creative Commons Attribution (CC BY) license (http://creativecommons.org/licenses/by/4.0/).
